# Impact of Obstetrical Emergency Code (Code Pink) Activation on Maternal and Neonatal Outcomes: A Retrospective Study

**DOI:** 10.7759/cureus.99302

**Published:** 2025-12-15

**Authors:** Israa AlMulai, Shalini Fernandes, Deba Nizami, Tasneem A Abuhajjaj, Reham Ainawi, Nancy Augustine, Daisy Verghese, Ayaz K Mallick

**Affiliations:** 1 Obstetrics and Gynaecology, Latifa Hospital, Dubai, ARE; 2 Obstetrics and Gynaecology, Mohammed Bin Rashid University of Medicine and Health Sciences, Dubai, ARE; 3 Obstetrics and Gynaecology, Dubai Medical College for Girls, Dubai, ARE; 4 Clinical Biochemistry, College of Medicine, King Khalid University, Abha, SAU

**Keywords:** cesarean section, code pink, decision-to-delivery interval, emergency medical services, nicu admission

## Abstract

Background

Obstetrical emergencies require rapid, coordinated responses to mitigate adverse maternal and neonatal outcomes. Hospitals use standardized emergency codes to ensure quick staff mobilization. Limited data exist on implementing obstetric emergency codes in the Middle East. This study evaluates the effectiveness of obstetrical emergency code activation (Code Pink) in a tertiary care hospital, focusing on its impact on maternal and neonatal outcomes.

Methodology

This retrospective cohort study was conducted in Latifa Women and Children Hospital, Dubai, UAE, a tertiary care center, between January 2020 and December 2022. The hospital records of 394 deliveries that required Code Pink activation were reviewed for maternal and neonatal outcomes. This retrospective, hospital-based study was conducted on a total of 381 participants. There were 12 cases of multiple pregnancies (one triplet and 11 twins); 394 deliveries were conducted. Key variables assessed included demographic data, obstetric history, indication for code pink, decision-to-delivery interval (DDI), mode of delivery, anesthesia used, blood loss, maternal complications, neonatal APGAR scores, umbilical cord pH, and NICU admission. Descriptive and inferential statistical analyses were performed using SPSS Statistics version 25.0 (IBM Corp., Armonk, NY, USA), and chi-square tests and t-tests were used to determine associations. A p-value < 0.05 was used as a threshold for significance.

Results

The mean age and BMI of the participants were 31.99 ± 6.12 years and 30.44 ± 5.45, respectively. Cesarean sections accounted for 90.1% (n=343) of deliveries, with general anesthesia used in 64.3% (n=245) of cases. The median APGAR scores were 7 at one minute and 9 at five minutes. Fetal distress was the leading indication for emergencies at 72.7% (n=245), followed by maternal causes at 15% (n=57). The NICU admission was observed in 46.4% (n=183) of neonates, with a statistically significant association between longer response times and higher NICU admissions (p < 0.001). Cesarean delivery was also significantly associated with increased NICU admissions (p = 0.024).

Conclusions

This study underscores the importance of code pink activation in urgent, life-saving obstetrical measures that improve mother and newborn outcomes. High-quality treatment requires improving training, simplifying methods, and using data-driven tactics. This study offers a foundation for future emergency obstetric care research and improvement.

## Introduction

Obstetric emergencies are critical medical conditions that occur during pregnancy, labor, or childbirth. These conditions include various complications like bleeding, pre-eclampsia, infection, obstructed labor, and issues stemming from miscarriage or abortion. Although many pregnancies and deliveries are uncomplicated, inherent dangers remain throughout the gestational period [1,2]. A seamless transition from intrauterine to extrauterine life necessitates a delicate equilibrium of maternal health, the progression of pregnancy, the delivery method, and early postpartum care. These emergencies have a detrimental impact on both the mother's and the neonate's health. According to current data provided by the World Health Organization, the worldwide maternal mortality rate (MMR) and neonatal mortality rate (NMR) are 223 per 100,000 live births and 17.3 per 1,000 live births, respectively. These mortality indicators are higher in resource-limited countries like the sub-Saharan region than in developed nations [3]. According to data from 2020, the UAE has an MMR of 9.34 per 100,000 live births and just under 3 neonatal deaths per 1000 live births [4]. The most common causes of maternal death are complications during the delivery, whereas fetal complications such as asphyxia, distress, and premature birth are some leading causes of newborn deaths [5].

To deal with emergencies, hospitals and healthcare facilities follow standardized emergency codes that allow quick responses among the hospital staff with minimal understanding [6,7]. In 2016, Latifa Hospital (Dubai, UAE) developed a multidisciplinary strategy named Code Pink, comprising obstetricians, nurses, neonatologists, anesthetists, and midwives as code responders. The policy of Code Pink was designed to outline the roles of responders, their responsibilities, and procedures for activating the code to handle obstetric emergencies promptly.

As in the case of any emergency code, a quick, prompt, and effective response is necessary for the successful outcome of Code Pink. Literature on successfully implementing obstetric emergency codes is limited, particularly in the Middle East. Moreover, no study has been done to assess its effectiveness since the implementation of Code Pink at our hospital. Hence, this study was conducted to analyze the indications of Code Pink and the outcome regarding the mode of delivery and maternal and fetal well-being.

## Materials and methods

Study design

This descriptive retrospective study was conducted at Latifa Women and Children Hospital, Dubai, UAE, a tertiary care center specializing in maternal and neonatal care. Ethical clearance was obtained from the Dubai Scientific Research Ethics Committee (approval no. 02/2004_30), following which the hospital records of all the pregnant women admitted from January 2020 to December 2022 were analyzed. 

Study population and data collection

The hospital's electronic medical records (Salama program) database of all the pregnant women admitted from January 2020 to December 2022 was searched and analyzed. Pregnant women who required activation of the emergency Code Pink in their peripartum period were included in the study. All deliveries that did not require Code Pink activation and those whose data were missing or incomplete were excluded from the study.

Based on the inclusion and exclusion criteria, the data of 112 women in 2010, 151 in 2021, and 131 in 2022 were included, bringing the sample size to 394, out of which there were 12 cases of multiple pregnancies, with one triplet and 11 twins. After considering the cases of multiple pregnancies, the total sample size included in the study was 381. Data on age, BMI, and obstetrical information such as gestational age, parity index, indication for activation of Code Pink, time of its activation, time of delivery, and mode of delivery were recorded. Data were also collected regarding events during and after labor, including the choice of anesthesia, venous and arterial cord pH, maternal blood loss during labor and postpartum, and any history of infection at the site of the cesarean incision. Indicators of fetal well-being included cardiotocography (CTG) monitoring during the intrapartum period and the appearance, pulse, grimace, activity, and respiration (APGAR) score at one minute, followed by a repeat score five minutes after birth, venous and arterial cord pH, and data on NICU admission were also obtained.

Statistical analysis

The data collected for the study included discrete, continuous, and nominal variables. Data was analyzed using SPSS Statistics version 25.0 (IBM Corp., Armonk, NY, USA). The continuous variables were expressed using the measure of central tendency (mean and median, wherever appropriate). The nominal and discrete data were expressed as frequency and percentages. Comparative analyses were done using statistical tools based on the objectives of the study and the type of variables. The chi-square test was used to study the association between nominal variables, and Cramer’s V test was used to study the strength of this association. For continuous variables, independent t-tests were conducted to compare the mean values between the groups. The data, tables, and graphs were created using Microsoft Excel (Microsoft Corp., Redmond, WA, USA). A p-value < 0.05 was used as a threshold for significance.

## Results

Demographics of participants

**Figure 1:**
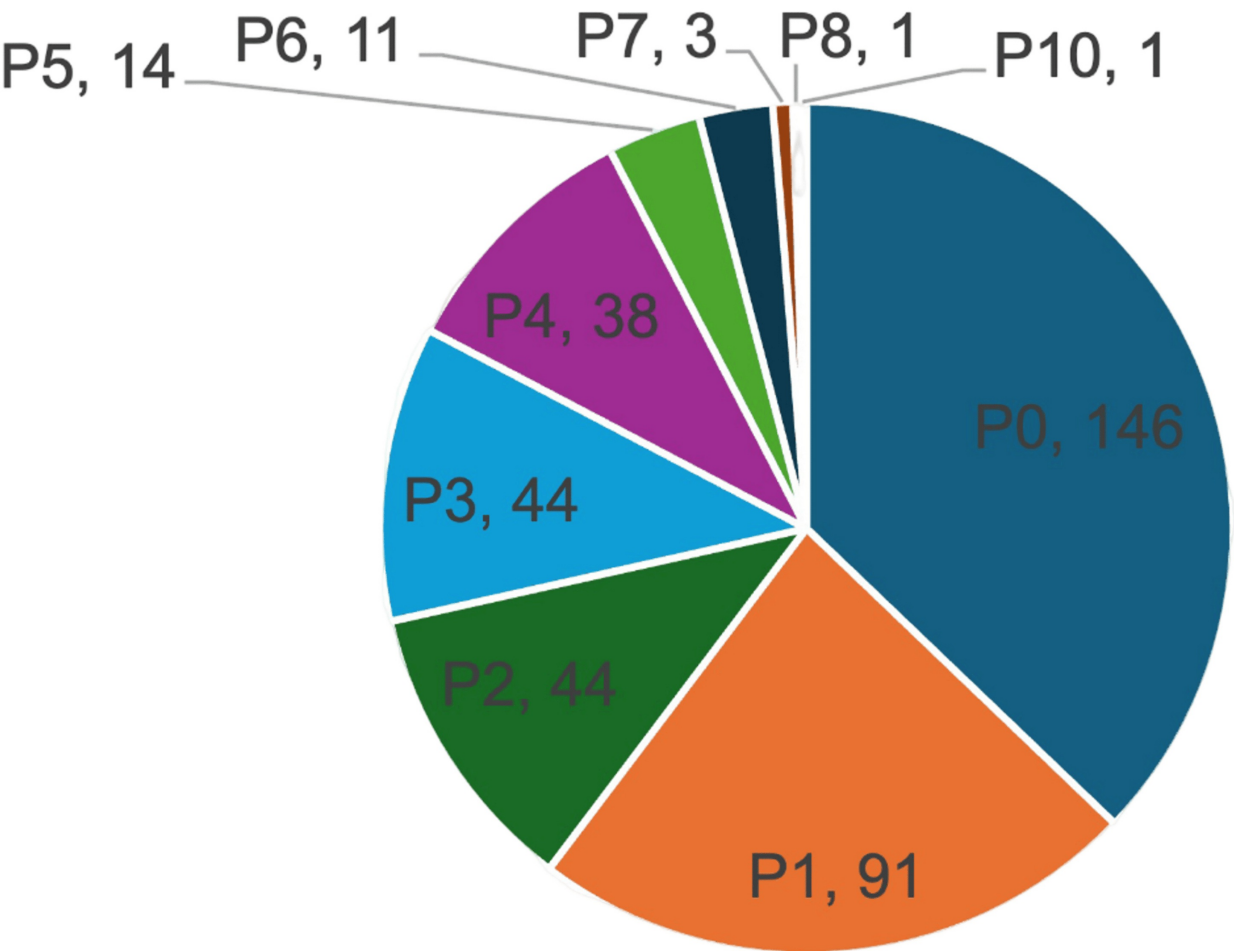
Pie chart showing the parity distribution of the participants (total n=381)

This retrospective hospital data-based study was conducted on a total of 381 participants having a mean age of 31.99 ± 6.12 years, ranging from 17 to 50 years (Table 1). Their mean BMI was 30.44 ± 5.45, with a range spanning from 19 to 56. Around 80.6% (n=307) were booked cases, whereas the rest, 19.4% (n=74), were not booked. Parity distribution revealed a predominance of nulliparous women of 37.3% (n=146), followed by a gradual decline to 23.4% (n=91) being P1, 11.3% (n=44) being P2, and only 0.3% (n=1) having a parity of P10 (Figure 1). The mean gestational age during which the obstetric emergencies were observed was 36.57 ± 3.98 SD. Once the emergency was announced, the average time to respond to the emergency was around 16.5 minutes (Table 2).

**Table 1:** Demographic details of the participants (total n=381)

Variables	Mean ± SD	Range
Age in years	31.99 ± 6.12	17 to 50
BMI	30.44 ± 5.45	19 to 56
Gestational age (weeks)	36.57 ± 3.98	23 to 45
Booking Status		
Booked	80.6 %	
Not booked	19.4 %	

**Table 2:** Average response time in minutes during obstetrical emergencies

Time interval (minutes)	Mean ± SD	Range
	16.5 ± 10	0 – 67

Maternal, obstetric, and neonatal outcomes

As there were 12 cases of multiple pregnancies (one triplet and 11 twins), a total of 394 deliveries were conducted. As seen in Table 3, most deliveries were conducted by cesarean section, accounting for 90.1% (n=343) of the cases. Around 7.1% (n=28) of deliveries were vaginal deliveries, followed by vacuum or forceps-assisted delivery at 2.5% (n=10). There was one single breech delivery, constituting only 0.3%. Regarding the type of anesthesia, a significant portion of around 64.3% (n=245) of patients underwent general anesthesia, followed by spinal and epidural anesthesia at 17.1% (n=65) and 10.2% (n=39), respectively. A small number of 8.4% did not require anesthesia (Table 3). Regarding blood loss during delivery, 82.1% (n=311) of women experienced total blood loss between 500 and 1000 ml, while blood loss of more than 1000 ml occurred in 9.5% (n=36) of cases. Surgical site infections were reported in 6.1% (n=23) of the participants (Table 3).

**Table 3:** Maternal and obstetrical outcomes among the participants

Outcome		Percentage
Mode of delivery	Vaginal delivery	27 (7.1%)
	Vacuum/forceps	10 (2.6%)
	Cesarean	343 (90.0%)
	Breech	1 (0.3%)
Type of anesthesia	General	245 (64.3%)
	Spinal	65 (17.1%)
	Epidural	39 (10.2%)
	None	32 (8.4%)
Total blood loss	Less than 500	32 (8.4%)
	500-1000	313 (82.1%)
	More than 1000	36 (9.4%)
Surgical site infection	Yes	23 (6.1%)
	No	358 (93.9%)

Following the delivery, the APGAR score was determined to assess the neonatal well-being. The median APGAR score at one minute was 7.0 (interquartile range: 5.0-8.0), while at five minutes, it improved to 9.0 (interquartile range: 8.0-9.0) (Table 4). The arterial cord blood collected during the intrapartum phase had a mean pH of 7.23 ± 1.05, while the venous cord blood pH was slightly higher at 7.25 ± 1.05. These ranges were within the expected physiological range (Table 4). The data from this study also indicated a significant post-delivery NICU admission rate, with approximately 46.4% (n=183) of neonates requiring intensive care support (Table 5).

**Table 4:** Indicators of neonatal outcomes, the APGAR score, and cord blood pH APGAR: Appearance, pulse, grimace, activity, and respiration

APGAR score	Median	Range (25^th^ to 75^th^ percentile)
At the end of 1 min	7.0	5.0 to 8.0
At the end of 5 mins	9.0	8.0 to 9.0
Cord blood pH	Mean	Range
Arterial cord blood	7.23 ± 1.05	6.77 to 7.44
Venous cord blood	7.25 ± 1.05	6.67 to 7.43

**Table 5:** Comparison of the admission of the neonates to the NICU and the time interval required to respond to the Code Pink

Parameter	NICU admission (group 1)	No NICU admission (group 2)	p-value
Number of deliveries	46.4 % (n=183)	53.6 % (n=211)	
Time interval for response to Code Pink	18.8 ± 14.1	15 ± 6.5	< 0.001

Indications for obstetrical emergency

The indications for obstetrical emergencies were categorized into fetal, maternal, and presentation-related causes. Fetal cause was the predominant cause for emergencies, accounting for 72.7% (n=277), followed by 15% (n=57) maternal causes. Malpresentation accounted for 9.2% (n=35), whereas the remainder were attributed to multiple pregnancies. Among the fetal causes, fetal distress was the most common indication (n=245), followed by cord prolapse (n=24) and shoulder dystocia (n=8). Antepartum causes such as antepartum hemorrhage (APH) (n=19) and abruptio placenta (n=16) were significant maternal indications for obstetrical emergencies. These findings highlight the significance of early diagnosis and management. The breech presentation was the most common malpresentation (n=32), underscoring the need for careful fetal assessment and consideration of appropriate delivery modes. Triplets were observed in one case, and twin pregnancies with complications, such as fetal distress, preterm labor, and APH, were noted in a few cases. Other causes for emergencies are summarized in Table 6.

**Table 6:** Frequency of the indication for activation of the emergency Code Pink APH: Antepartum hemorrhage, DCDA: Dichorionic diamniotic

Causes	Indications	N
Fetal causes	Fetal distress	245
	Cord prolapse	24
	Shoulder dystocia	8
Maternal causes	APH	35
	Previous CS and related complications	12
	Preeclampsia and eclampsia	6
	Failure to progression of labour	2
	Postpartum hemorrhage	2
Multiple pregnancies	Triplets preterm in labour	1
	Twins with fetal distress	2
	Twins with breech/footling breech	3
	Twins-preterm labour	1
	Compound presentation of the second twin	1
	Severe sepsis/DCDA twins	1
	DCDA breech in labour	1
	Twins with APH	2
Malpresentation	Breech in labor	32
	Transverse lie in labour	2
	Malpresentation	1

Association between Code Pink response time and NICU admission

To study the effect of response time and the outcome of NICU admission, the total number of deliveries was categorized into two groups. Group I included newborns admitted to the NICU, and group 2 included babies not admitted to the NICU (Table 5). The average duration to response for NICU-admitted newborns was 18.5 ± 14 minutes compared to 15 ± 6.5 minutes for non-NICU-admitted neonates. An independent sample t-test was used to study the significance, and this difference in response time was statistically significant (p < 0.001).

Association between NICU admission with mode of delivery

A chi-square test was performed to assess the association between NICU admission of the neonate and the mode of delivery in case of an obstetrical emergency. As seen in Tables 7-8, the newborns of 48.5% (n=172) of women who underwent cesarean section were more likely to be admitted to the NICU compared to 35.7% of those who had vaginal delivery (n=10). This observation was statistically significant (p = 0.024).

**Table 7:** Comparison of neonatal outcomes based on NICU admission across different delivery methods. The data are presented as frequency and percentage.

Mode of delivery	NICU admission n (%)	No NICU admission, n (%)	Total n (%)
Cesarean	172 (43.7%)	183 (46.4%)	355 (90.1%)
Vaginal	10 (2.5%)	18 (4.6%)	28 (7.1%)
Vacuum/forceps	1 (0.3%)	9 (2.3%)	10 (2.5%)
Breech	0 (0.0%)	1 (0.3%)	1 (0.3%)
Total	183 (46.4%)	211 (53.6%)	394 (100%)

**Table 8:** Data showing the chi-Square test and likelihood ratio for newborns admitted to the NICU following emergency cesarean sections due to obstetrical emergencies A p-value <0.05 was considered significant.

Test	Value	p-value
Pearson chi-square	8.073	0.044
Likelihood ratio	9.415	0.024

## Discussion

The study examined maternal, obstetric, and neonatal outcomes in cases requiring emergency Code Pink activation in a tertiary care center in Dubai. By analyzing data from 381 participants, we gained valuable insights into demographics, outcomes, and factors influencing neonatal and maternal well-being during obstetric emergencies.

The mean age of the study participants was 31.99 ± 6.12 years, with a significant proportion being nulliparous (37.3%). The mean BMI of 30.44 ± 5.45 suggests a notable prevalence of overweight or obesity, which is consistent with the UAE population data [4]. This is a relevant finding, as obesity is associated with increased obstetric risks, including fetal distress and complications during labor [1,8]. The high proportion of booked cases (80.6%) reflects a well-organized antenatal care system, which aligns with findings from other tertiary care centers globally [1,9].

The study revealed a cesarean section rate of 90.1%, significantly higher than the global average of 21.1% [3]. This is likely due to the emergency nature of the cases, as obstetric emergencies often necessitate surgical intervention to prevent adverse maternal or neonatal outcomes. The high proportion of general anesthesia use (64.3%) may be attributed to the urgent nature of Code Pink activations, where speed often takes precedence over anesthetic preference.

Fetal distress was the predominant reason for emergency activation, accounting for 72.7% of cases. This aligns with the findings by Heale et al. [8] and Taras et al. [9], who highlighted fetal distress as the leading cause of obstetric emergencies globally. Other fetal-related causes, such as cord prolapse and shoulder dystocia, underscore the need for rapid diagnosis and intervention to prevent adverse outcomes. The remaining indications were malpresentation (mainly breech presentation) and multiple gestation, which highlight the importance of regular antenatal follow-up and determination of mode of delivery in advance. However, in relation to our study population, many of the cases were unbooked in our facility, and the diagnosis was mostly done at the time of presentation. Among maternal causes, APH and abruptio placentae were significant contributors. These findings emphasize the importance of antenatal screening and preparedness to manage conditions like APH and placental abruption, which are associated with high maternal and neonatal morbidity [6,10].

Despite timely interventions, neonatal outcomes demonstrated challenges. Nearly 46.4% of neonates required NICU admission, and the mean cord blood pH values (7.23 arterial, 7.25 venous) were within the physiological norms. However, few studies have indicated altered cord blood pH, pointing toward a potential perinatal asphyxia [1,11]. These findings underscore the critical role of swift emergency responses in minimizing neonatal morbidity and mortality.

The study showed a strong association between NICU admissions and maternal blood loss, with hemorrhage (≥1000 mL) being a significant predictor (P < 0.001). This aligns with prior research indicating that maternal hemorrhage adversely affects neonatal outcomes due to compromised uteroplacental perfusion [12,13]. Surgical site infections, though observed in only 6% of cases, further underscore the need for robust infection control protocols.

The statistically significant delay in Code Pink response time for neonates admitted to the NICU (18.5 ± 14 minutes vs. 15 ± 6.5 minutes; p < 0.001) highlights the critical importance of rapid response systems in obstetrics. Studies by Nilakantam et al. [6] and Li et al. [14] corroborate that shorter decision-to-delivery intervals (DDI) can improve neonatal outcomes significantly. These findings reinforce the need for continuous optimization of response protocols to reduce delays. The analysis revealed a higher likelihood of NICU admission among neonates delivered via cesarean section (48.5%) compared to vaginal delivery (35.7%), with statistical significance (p = 0.024). While cesarean delivery is often necessary in emergencies, the association with adverse neonatal outcomes, including higher NICU admission rates, is well-documented [11,15].

The findings observed in this study can have several implications in clinical practice. First, a statistically significant association between response time and neonatal outcomes emphasizes the need for regular drills and protocol optimization to minimize delays. Hence, it can improve the emergency protocol. Second, antenatal care interventions, enhanced screening, and management of high-risk pregnancies, particularly in women with obesity and other comorbidities, could reduce the need for emergency interventions. Moreover, a multidisciplinary care team based on the integration of a rapid response team specializing in obstetrics could enhance maternal and neonatal outcomes in emergencies.

This study has its limitations. As a retrospective study, it is subject to potential biases, including incomplete or missing data. Additionally, the study's setting in a single tertiary care center may limit the generalizability of the findings. Another limitation is the absence of a control group, as this study is a single-arm study involving observational analysis of pregnant women who developed severe complications during delivery. This was because including a parallel group of pregnant women with serious conditions not receiving Code Pink intervention was practically and ethically not feasible. However, to address this limitation, the findings of this study were compared with those of other studies conducted on similar lines.

## Conclusions

This study highlights the effectiveness of Code Pink in managing obstetric emergencies and its significant impact on maternal and neonatal outcomes. The findings highlight areas for intervention, particularly in reducing response times and optimizing the management of maternal and neonatal complications. Continued emphasis on training, protocol refinement, and data-driven adjustments will be essential to further improve outcomes and ensure the highest standards of care for mothers and their newborns.
